# Addressing child undernutrition in Tanzania with the ASTUTE program

**DOI:** 10.1186/s40795-022-00511-0

**Published:** 2022-04-07

**Authors:** E Beckstead, G Mulokozi, M Jensen, J Smith, M Baldauf, K. A. Dearden, M. Linehan, S. Torres, J. Glenn, J. H. West, P. C. Hall, B. T. Crookston

**Affiliations:** 1 Brigham Young University, Provo, USA; 2 ASTUTE Program, IMA World Health, Dar es Salaam, Tanzania

**Keywords:** Behavior change communication, Child undernutrition, Infant and young child feeding practices, Mass media intervention

## Abstract

**Background:**

Optimal infant and young child feeding practices (IYCFP) reduce childhood stunting and are associated with additional health benefits. In Tanzania, IYCFP are far from optimal where 32% of children under the age of 5 years are stunted. The purpose of this study was to examine whether behavior change communication focused on reducing child undernutrition was associated with improved IYCFP in Tanzania.

**Methods:**

A cross-sectional survey was administered to approximately 10,000 households with children under the age of 2 at baseline and endline. Bivariate analyses and logistic regression was used to examine the relationship between exposure to behavior change communication and timely initiation of breastfeeding, exclusive breastfeeding, continued breastfeeding at one year, timely complementary feeding (CF), minimum meal frequency (MMF), minimum dietary diversity (MDD), and minimum acceptable diet (MAD).

**Results:**

Mothers who heard a radio spot about IYCFP were more likely than mothers who had not heard a radio spot about IYCFP to begin complementary foods at six months. Their children were also more likely to achieve MMF, MDD, and MAD with odds ratios of 2.227 (*p* = 0.0061), 1.222 (*p* = 0.0454), 1.618 (*p* =  < .0001), and 1.511 (*p* = 0.0002), respectively. Mothers who saw a TV spot about IYCFP were more likely to have greater odds of knowing when to begin complementary feeding, feeding their child a minimally diverse diet (4 food groups or more), and serving a minimum acceptable diet with odds ratios of 1.335 (*p* = 0.0081),

1.360 (*p* = 0.0003), and 1.268 (*p* = 0.0156), respectively.

**Conclusion:**

Exposure to behavior change communication in Tanzania was generally associated with some increased knowledge of optimal IYCFP as well as practicing IYCF behaviors. Behavior change communication planners and implementers may want to consider conducting similar campaigns as an important component of behavior change to reduce undernutrition and poor health outcomes in developing settings.

## Background

Evidence-based Infant and Young Child Feeding Practices (IYCFP) are defined as essential nutritional behaviors for children under the age of 2 and are an effective intervention used to improve children’s health [1]. These practices include breastfeeding within the first hour of life, breastfeeding exclusively for the first six months of life, beginning complementary feeding at 6–8 months, feeding children diverse diets, and feeding children at least the minimum number of times per day (minimum meal frequency, MMF) [2].

Sub-optimal IYCFP presents significant challenges and is a public health concern in Tanzania. Chronic undernutrition under the age of 5 is associated with less schooling, late entry into starting school, and between 22 to 45% less income throughout a lifetime [3]. Tanzania has faced low rates of optimal IYCFP for many years. One study indicates that only 46.1% of mothers in Tanzania initiated breastfeeding within the first hour, and 41% to 49.9% of infants under the age of 6 months were exclusively breastfed [4, 5]. Additionally, about half (49.3%) of children 6 to 23 months of age had MDD, 70.3% met the standard for MMF [4], and 30.1% received a MAD [6]. MDD is defined as the consumption of foods from at least 4 food groups within a period of 24 h [7].

Failure to provide a child with optimal IYCFP is associated with stunting and severe illness [1]. Stunting in children is illustrated by low-height-for-age; stunted individuals are much shorter than their healthy peers, even years later [1]. Other long-term effects of stunting include permanently reduced cognitive and motor development, increased risk of degenerative diseases, premature death, poor performance in school, and decreased economic productivity [8–10]. About 32% of children under the age of 5 in Tanzania are stunted [6]. Tanzania’s National Nutrition plan aims to reduce stunting to 28% by 2021 [6]. By 2019, substantial progress had been made towards this goal, though an estimated 2,700,000 children under 5 years remained stunted [6].

While integrated communication and capacity building programs are a strategy often utilized to disseminate information, shape norms, and influence a mother's nutrition and feeding practices [11], media-based efforts are often used to create awareness of local issues and generate attention from the community [11]. Literature indicates that programs which incorporate various communication strategies play a central role in addressing the nutrition of young children in Tanzania [12]. While not in Tanzania, one study analyzed the combined impact of integrated programs using capacity building, mass media interventions, community mobilization, and interpersonal communication (IPC) on breastfeeding and complementary feeding (CF) practices [7]. This large-scale study conducted in Vietnam, Ethiopia and Bangladesh found that CF indicators improved over time among both groups: those exposed to all 3 interventions (IPC, mass media, community mobilization), and those exposed to standard IPC but less intensive mass media and community mobilization [7]. However, there was no differential improvement among the group that was more intensively exposed to mass media and community mobilization [7]. Specifically relating to Tanzania, studies have confirmed the association between dietary diversity and reduction of undernutrition among children and others have suggested that behavioral change communication strategies may help reduce rates of undernutrition. However, studies using large-scale data from Tanzania to examine the direct association between mass media and IYCFP have not been identified in literature [12, 13].

The purpose of this study was to examine the relationship between behavior change communication and critical child nutrition and health behaviors in Tanzania. Specific research questions included:

- Did parental knowledge about and behaviors of IYCFP change over time before and after the behavior change communication was implemented?
- Is communication campaign exposure associated with increased parental knowledge about IYCFP?
- Is communication campaign exposure associated with increased infant and young child feeding practices? (i.e. MMF, diet diversity, initiation of breastfeeding within 24 h, exclusive breastfeeding for the first 6 months, complementary feedings starting at 6–8 months)

## Methods

### Study design

From 2015–2020, IMA World Health (IMA) implemented the Department for International Development-funded “Scaling up Growth: Addressing Stunting in Tanzania Early (in the under 5’s)” (ASTUTE). The project was designed in close collaboration with the Tanzanian Ministry of Health. ASTUTE was specifically designed to support the Tanzania’s nutrition strategy, including a mandate to build the capacity of District Nutrition Officers (DNuOs) to manage and coordinate, at the district level, the nine nutrition-relevant government sectors through Council Multisectoral Steering Committees for Nutrition (CMSCN), mirroring the coordination at the national level by the High Level Steering Committee on Nutrition (HLSCN). The National Institute for Medical Research provided ethical clearance.

**Fig. 1:**
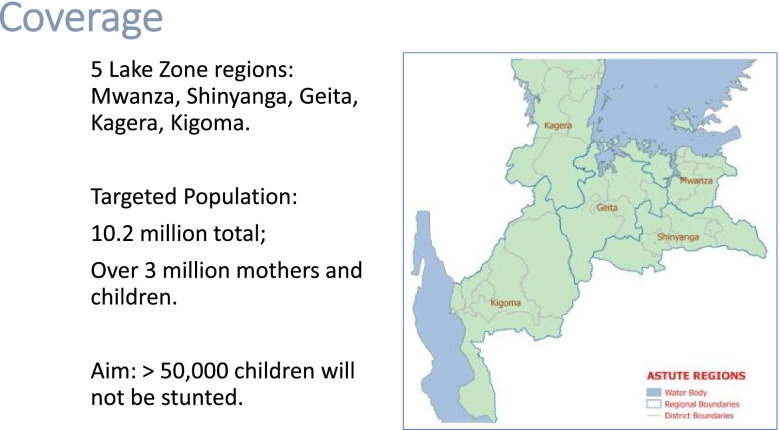
Map of intervention area. This map was provided by the ASTUTE program and is used with permission

The behavior change communication was implemented in five regions of the Lake Zone in Tanzania (see Fig. 1). These are Geita, Kagera, Kigoma, Mwanza, and Shinyanga with a collective population of 10.2 million and over 750,000 stunted children. These regions were selected for their documented high prevalence of stunting and anemia and poor infant and child feeding. The behavior change communication focused on three major objectives:

1. Building capacity of the local government to implement and manage high quality nutrition-specific and nutrition-sensitive activities to reduce childhood stunting, including facility-based nutrition services and community out-reach by community health workers (CHWs), through joint planning, skill training, on-going mentoring and performance monitoring interventions to prioritize and allocate district resources for coordinated nutrition activities, supplies, and messages.
2. Increasing the knowledge of mothers and caregivers to develop a new understanding of what children need to eat to thrive, and to adopt and support improved child feeding practices. This was implemented by CHWs and volunteers working with community-based organizations who mobilized communities and conducted home visits using the negotiating for behavior change strategy; carried out support groups for mothers, male head of households, and other caregivers; and implemented the positive deviance/hearth approach to rehabilitating malnourished children. Additionally, ASTUTE increased mothers’ and caregivers’ knowledge through large-scale radio and TV campaigns implemented for the duration of the project. Messaging for all activities revolved around stunting prevention practices including early and exclusive breastfeeding, appropriate complementary feeding, hand hygiene and sanitation, early childhood development, and men’s support for women during pregnancy and post-delivery.
3. Increasing the knowledge of adolescent girls, reproductive age women, mothers, caregivers, households and community decision makers through facility-based health promotion and community and household-based

In the designated regions, an evidence-based communication campaign was implemented between June 2017 and March 2020, which included radio and TV campaigns (17.6 million reached), mobile outreach (8.4 million reached through direct messaging), and IPC in the form of home visits (6.4 million reached). The radio spots were theory-based and lasted 60 s. They were broadcast 10 times a day for a total of 70,000 times. TV spots were also 60 s and aired a total of 1, 198 times. They aired on three different national/regional stations before and during the news.

A mother’s knowledge about IYCFP, as well as environmental and social influences are important determinants of her nutrition related behaviors. Social Cognitive Theory (SCT) is a well-established theoretical approach that may be utilized to address parental feeding practices and to inform behavior change communication development [14]. The constructs of SCT are cognitive influences, environmental influences, and supporting behavioral factors such as self-efficacy [15]. Many communication campaigns utilize techniques from SCT by modeling desired behaviors on television using actors that are culturally or ethnically similar to the audience [11]. When done correctly, behavior change strategies can help increase the self-efficacy of the audience, and can address inappropriate cultural or social practices that adversely affect childhood nutrition status [11, 12]. The use of SCT allows for a more thorough understanding of nutrition-related behaviors [16, 17].

### Sampling

The ASTUTE program utilized a cross-sectional survey that was distributed to 5,000 households before the behavior change communication was implemented and an additional 4,996 households after the behavior change communication was implemented. Inclusion criteria included having a child under two years (0–23 months) of age and living in the regions where the campaign took place. Respondents who did not meet these criteria were excluded from the study. Survey questions were directed to the female caregiver of the youngest child in the household, and if available, to the male head of household. Consent was received before the survey was administered, and participants understood that their participation was voluntary, they could stop at any time, and the potential risks and benefits associated with their participation. The survey was developed in English, and then translated to Swahili. It was piloted and edited, and ultimately included 169 questions which aimed to measure participants’ exposure to the communication campaign and other outcomes.

### Procedure

Data were collected by a field team consisting of 50 enumerators and 10 supervisors. All field team members received a two-week training prior to participant recruitment and data collection. Their goal was to recruit 5,000 households during three rounds of surveys. Survey participants were selected using a stratified, multi-stage random sample design. During the baseline round, 243 villages were included and participants were randomly sampled within each village. During the following two rounds of surveys the same villages were used, but participants were again randomly selected. Interviews were conducted in the participants’ homes and lasted on average 50–60 min. Data were collected digitally using smartphones and PDAs (personal digital assistants).

At baseline, 5,000 female caregivers and 1,114 male heads of household were surveyed from January to February 2017. At endline, 4,993 female caregivers and 3,084 male heads of household were surveyed from January to February 2020. While the survey used in this study was not validated as part of this work, it was largely based on previously validated instruments such as the Demographic and Health Surveys and was pilot tested in the field before baseline data were collected. Behavior change communication objectives analyzed during the study included the reach and exposure of the campaign, change of key indicators, and the association between exposure and key indicators.

Authorization for this research and intervention was obtained from the Ministry of Health in Tanzania and Development Media International’s (DMI) internal IRB. Data quality was checked by 11 controllers, and if the quality of a previously completed interview could not be validated, a new interview was conducted. Additionally, raw data was checked for outliers and invalid answers.

### Measurement

Female caregivers provided demographic and household information in the survey to understand the distribution in each sample group. Before data analysis occurred, a single binary definition was created for each variable of the campaign. Primary and secondary outcome variables were also defined for each campaign message theme.

A wealth index variable was created to estimate relative household income. This variable was based on the index created by Briones [18]. It includes household access to services such as safe water and sanitation as well as ownership of goods including radio, TV, bicycle, motorcycle, automobile, mobile phone, boat, and animal-drawn cart. The score is the average of the services and goods scores. Values range between 0 and 1, with wealth increasing as the value gets closer to 1.

Two female and two male media exposure variables were created. Female exposure to radio was defined as ‘yes’ if women reported recalling a radio message that discussed maternal nutrition, exclusive breastfeeding, or child nutrition after six months. Female exposure to TV was defined as ‘yes’ if women reported recalling a TV message about maternal nutrition, exclusive breastfeeding, or child nutrition after six months. TV and radio variables were also created for males using the same methodology.

A variable was created to measure overall exposure to the behavior change communication. This variable, which only includes responses from mothers, assesses whether the respondent had no exposure to the behavior change communication, heard or watched any behavior change messages (media only), had any IPC-related interactions (IPC only), or both media and IPC.

Seven variables measuring feeding practices were used, in alignment with World Health Organization (WHO) standards for IYCF indicators [10]. They included timely initiation of breastfeeding, exclusive breastfeeding, continued breastfeeding at one year, timely complementary feeding (CF), minimum meal frequency (MMF), minimum dietary diversity (MDD), and minimum acceptable diet (MAD). These variables are described below.

#### Timely initiation of breastfeeding

Timely initiation of breastfeeding, as defined by the WHO, means beginning breastfeeding within the first hour of life [10]. Putting baby to breast immediately or within the first hour was considered timely. The question used to create this variable was “how long after birth did you first put (name) to breast?”.

#### Exclusive breastfeeding

For exclusive breastfeeding, the primary outcome was defined as “proportion of mothers of children 0–6 months who are currently breastfeeding and report they haven’t given the child any other food/liquids.” This was assessed by asking participants whether they are breastfeeding and to select food and drinks they had fed their infant within the last 24 h.

#### Continued breastfeeding at one year

Continued breastfeeding at one year was assessed by asking mothers of infants ages 12–15 months whether they are “still breastfeeding (name).”

#### Timely Complementary Feeding (CF)

For complementary feeding, mothers of children ages 6–8 months were asked: “Have you introduced (name) any other fluids or foods besides breast milk?” “How old was (name) when he/she was first fed something other than breast milk?”.

#### Minimum Meal Frequency (MMF)

MMF is defined by the WHO as being fed 2 times per day for breastfed infants 6–8 months, 3 times for breastfed children 9–23 months, and 4 times for non-breastfed children 6–23 months [2]. Based on these standards, participants were asked the age of their child and whether they are breastfed. They were then asked if the child ate anything when they woke up in the morning, anything between then and lunch, anything at lunch, anything between lunch and dinner, anything at dinner, and anything after dinner.

#### Minimum Dietary Diversity (MDD)

This variable measured how many food groups were represented in the child’s (6–23 months) diet in the previous day. Participants were asked to select whether their child had eaten specific types of grains, legumes, dairy, flesh, eggs, and fruit/vegetables. Children who ate from four or more of these groups were coded as having MDD.

#### Minimum Acceptable Diet (MAD)

If children ages 6–23 months achieved MDD and MMF, then they were coded as having a MAD.

### Analysis

The raw data were cleaned, recoded if necessary, and analyzed using SAS version 9.4. The baseline data were first compared to the endline data to determine change for the key indicators.

A chi-square test was conducted to determine whether these differences in key indicators.

between baseline and endline were statistically different. A multiple logistic regression model was built to determine the relationship between exposure to the media campaign and increased IYCFP among female caregivers. The model controlled for maternal and male head of household age, education, child age, and wealth.

## Results

There were 9,996 female survey participants (combining those at baseline and at endline) who answered questions (see Table 1). Demographic characteristics were relatively consistent from baseline to endline. Most respondents reported living in a rural setting (86%) with crop farming as the main occupation (70.77%). Most were Christian (83.32%) and were in a monogamous marriage (74.02%). All questions regarding feeding practices related to those who had children between 0 and 24 months with the average age of child in that demographic being 9.5 months.

**Table 1:** Demographics

Description	Baseline (percent)	Endline (percent)	Combined total
**Female caregivers surveyed**	**5000**	**4996**	**9996**
**Setting**			
Rural	4301 (86.02)	4296 (85.99)	8,597 (86.00)
Urban	699 (13.98)	700 (14.01)	1,399 (14.00)
**Religion**			
Christians	4166 (83.32)	–	4,166(83.32)
Muslim	541 (10.82)	–	541 (10.82)
Other religion	102 (2.04)	–	102 (2.04)
No religion	191 (3.82)	–	191 (3.82)
**Marital Status**			
Single—never married	293 (5.86)	211 (4.22)	504 (5.04)
Married—monogamous	3549 (70.98)	3850 (77.06)	7399 (74.02)
Married – polygamous	291 (5.82)	490 (9.81)	781 (7.81)
Informal union	425 (8.50)	75 (1.50)	500 (5.00)
Single – Previously married	442 (8.84)	365 (7.3)	807 (8.07)
**Occupation**			
Crop farming	3576 (71.52)	3498 (70.02)	7074 (70.77)
Self-employed	386 (7.72)	699 (13.99)	1085 (10.85)
Housewife/ Househusband	755 (15.10)	605 (12.11)	1360 (13.61)
Other	278 (6.7)	194 (3.88)	472 (4.72)
**Can read**			
Yes	3684 (73.68)	3903 (78.17)	7587 (75.92)
No	1285 (25.70)	1090 (21.83)	2375 (23.77)
**Education**			
Less than primary school	1546 (30.92)	1481 (29.65)	3027 (30.29)
Completed primary school	2808 (56.16)	2840 (56.86)	5648 (56.51)
Some secondary education or more	646 (12.92)	674 (13.49)	1320 (13.21)
** Mean wealth**^a^	0.314 (SD 0.185)	0.364 (SD 0.176)	0.338 (SD 0.182)
** Mean age of child (0–23 months) in months**	9.38 (SD 6.60)	9.68 (SD6.70)	9.51 (SD 6.64)
** Male heads of household surveyed**	1,114	3,084	4,198
**Education**			
Less than primary school	215 (18.79)	–	–
Completed primary school	751 (65.65)	–	–
Some secondary education or more	178 (15.56)	–	–

^a^statistically significant

Note: The wealth variable was created by combining two other indices: access to services (access to water and safe sanitation) and ownership of eight specific consumer durables (TV, radio, automobile, etcetera). It is an average of these two and assumes they are equal. The result is a value between 0 and 1, a score closer to 1 indicating a higher socio-economic status [17]

While baseline data were not available for all variables, at the end of the campaign, nearly all reported behaviors had increased at endline (see Table 2). Endline data demonstrate compliance was high for timely initiation of breastfeeding (83.62%), exclusive breastfeeding (83.25%), timely complementary feeding (77.62%) and continued breastfeeding through 1 year of age (90.79%).

**Table 2:** IYCF behaviors frequency baseline vs. endline

Variable	Baseline % (n)	Endline % (n)	Chi Square
**Key Knowledge Indicators**			
Knowledge of when to initiate breastfeeding	82.98 (4149)	90.12 (4417)	< .0001
Knowledge of when to start complementary feeding	72.66 (3633)	87.21 (4349)	< .0001
**Key Behavior Indicators**			
Timely initiation of breastfeeding	64.53 (2963)	83.62 (4008)	< .0001
Exclusive breastfeeding	28.69 (503)	83.25 (1426)	< .0001
Timely complementary feeding	60.19 (375)	77.62 (496)	< .0001
Continued breastfeeding at 1 year	81.76 (632)	90.79 (739)	< .0001
Minimum meal frequency (MMF)	–––––––-	70.33 (4326)	N/A
Minimum dietary diversity (MDD) for child (4 food groups)	–––––––-	47.46 (1558)	N/A
Minimum acceptable diet (MAD)	–––––––-	22.34 (717)	N/A

Mothers who recalled hearing a radio ad about IYCFP were more likely to practice key IYCFP behaviors and have knowledge than those who had not heard radio ads (see Table 3). For example, mothers who recalled hearing a radio ad about IYCFP had greater odds of achieving timely initiation of CF at six months, MMF, MDD, and MAD.

**Table 3:** Regression analysis for maternal exposure to radio IYCFP Intervention and key IYCF knowledge and practice

**Key Knowledge Indicators**	**Odds Ratio**	**Confidence Interval**
Knowledge of when to initiate breastfeeding	0.682	0.536–0.868^a^
Knowledge of when to initiate complementary feeding	1.183	0.923–1.516
**Key Behavior Indicators**	**Odds Ratio**	**Confidence Interval**
Timely initiation of breastfeeding	1.051	0.850–1.300
Exclusive breastfeeding	1.004	0.703–1.432
Timely complementary feeding	2.227	1.256–3.948^a^
Continued breastfeeding at 1 year	1.488	0.789–2.805
Minimum meal frequency	1.222	1.004–1.486^a^
Minimum diversity for child (4 food groups)	1.618	1.328–1.972^a^
Minimum acceptable diet	1.511	1.213–1.883^a^

^a^statistically significant

Note: Multivariate logistic regression model predicting key IYCFP knowledge and practices using intervention exposure: whether the mother saw a TV commercial or heard a radio spot promoting key IYCFP messages from the behavior change communication. Each indicator estimate is derived from a multivariate analysis that estimates the probability of the health indicator given exposure to the intervention. All model estimates control for maternal age, maternal education level, and wealth index

Male head of household exposure to radio ads was not as often associated with higher knowledge or behaviors as was maternal exposure (see Table 4). Specifically, male heads of household who recalled hearing a radio ad about IYCFP had greater odds of the mother knowing when to initiate CF and practicing timely initiation of breastfeeding.

**Table 4:** Regression analysis for male head of household’s IYCFP exposure to radio intervention and maternal IYCF knowledge and practice

**Key Knowledge Indicators**	**Odds Ratio**	**Confidence Interval**
Knowledge of when to initiate breastfeeding	1.455	1.087–1.949^a^
Knowledge of when to initiate complementary feeding	1.192	0.930–1.529
**Key Behavior Indicators**	**Odds Ratio**	**Confidence Interval**
Timely initiation of breastfeeding	1.379	1.072–1.772^a^
Exclusive breastfeeding	0.703	0.486–1.017
Timely complementary feeding	0.946	0.554–1.616
Continued breastfeeding at 1 year	1.113	0.546–2.268
Minimum meal frequency (MMF)	1.181	0.966–1.443
Minimum dietary diversity (MDD) for child (4 food groups)	0.979	0.802–1.195
Minimum acceptable diet (MAD)	1.167	0.923–1.477

^a^statistically significant

Note: Multivariate logistic regression model predicting key IYCFP knowledge and practices using intervention exposure: whether the male head of household saw a TV commercial or heard a radio spot promoting key IYCFP messages from the behavior change communication. Each indicator estimate is derived from a multivariate regression model that estimates the probability of the health indicator given exposure to the intervention. All model estimates control for maternal age, maternal education level, and wealth index

Maternal exposure to TV ads focused on key child messages was associated with greater knowledge and behavior in a few instances (see Table 5). Examples include knowledge of CF, feeding children a diverse diet (4 food groups or more), and serving a MAD.

**Table 5:** Regression analysis for maternal exposure to IYCFP TV messages and IYCF knowledge and practice

**Key Knowledge Indicators**	**Odds Ratio**	**Confidence Interval**
Knowledge of when to initiate breastfeeding	0.872	0.702–1.084
Knowledge of when to initiate complementary feeding	1.335	1.078–1.652^a^
**Key Behavior Indicators**	**Odds Ratio**	**Confidence Interval**
Timely initiation of breastfeeding	1.130	0.940–1.360
Exclusive breastfeeding	0.791	0.585–1.068
Timely complementary feeding	1.206	0.750–1.938
Continued breastfeeding at 1 year	0.950	0.545–1.657
Minimum meal frequency (MMF)	1.021	0.864–1.207
Minimum dietary diversity (MDD) for child (4 food groups)	1.360	1.151–1.607^a^
Minimum acceptable diet (MAD)	1.268	1.046–1.537^*^

^a^statistically significant

Note: Multivariate logistic regression model predicting key IYCF knowledge and practices using intervention exposure: whether the mother saw a TV commercial or heard a radio spot promoting key IYCFP messages from the behavior change communication. Each indicator estimate is derived from a multivariate logistic regression model that estimates the probability of the health indicator given exposure to the intervention. All model estimates control for maternal age, maternal education level, and wealth index

Similar to maternal exposure, when male heads of household reported exposure to these TV advertisements their child had greater odds of having a diverse diet and a MAD. However, male heads of household who recalled seeing these messages had lower odds of the mother knowing when to begin breastfeeding (see Table 6).

**Table 6:** Regression analysis for male head of household’s exposure to IYCF TV messages and maternal key IYCF knowledge and practice

**Key Knowledge Indicators**	**Odds Ratio**	**Confidence Interval**
Knowledge of when to initiate breastfeeding	0.659	0.505–0.860^a^
Knowledge of when to initiate complementary feeding	0.974	0.760–1.249
**Key Behavior Indicators**	**Odds Ratio**	**Confidence Interval**
Timely initiation of breastfeeding	0.976	0.771–1.234
Exclusive breastfeeding	1.141	0.778–1.676
Timely complementary feeding	0.829	0.468–1.466
Continued breastfeeding at 1 year	0.593	0.302–1.163
Minimum meal frequency (MMF)	1.087	0.884–1.337
Minimum dietary diversity (MDD) for child (4 food groups)	1.495	1.217–1.836^*^
Minimum acceptable diet (MAD)	1.455	1.147–1.845^a^

^a^statistically significant

Note: Multivariate logistic regression model predicting key IYCF knowledge and practices using intervention exposure: whether the male head of household saw a TV commercial or heard a radio spot promoting key IYCFP messages from the behavior change communication. Each indicator estimate is derived from a multivariate logistic regression model that estimates the probability of the health indicator given exposure to the intervention. All model estimates control for maternal age, maternal education level, and wealth index

The association between overall behavior change communication exposure, and/or IPC, and knowledge and behaviors of mothers related to IYCFP showed mixed results (see Table 7).

**Table 7:** Regression analysis for maternal exposure to behavior change communication and key IYCF knowledge and practice

**Key Knowledge Indicators**	No Exposure OR (CI)	Media Only OR (CI)	IPC Only OR (CI)	Media + IPC OR (CI)
Knowledge of when to initiate breastfeeding	–	0.74 (0.48, 1.15)	0.68 (0.54, 0.86)^a^	0.60 (0.44, 0.81)^a^
Knowledge of when to initiate complementary feeding	–	0.94 (0.65, 1.36)	1.04 (0.85, 1.26)	0.91 (0.70, 1.20)
**Key Behavior Indicators**				
Timely initiation of breastfeeding	–	0.82 (0.58, 1.17)	0.75 (0.63, 0.90)^a^	1.00 (0.77, 1.31)
Exclusive breastfeeding	–	1.08 (0.56, 2.07)	0.90 (0.66, 1.23)	0.57 (0.38, 0.85)
Timely complementary feeding	–	0.63 (0.25, 1.59)	1.06 (0.68, 1.68)	1.10 (0.56, 2.14)
Continued breastfeeding at 1 year	–	4.38 (0.57, 33.58)	0.93 (0.53, 1.64)	1.40 (0.62, 3.16)
Minimum meal frequency (MMF)	–	1.11 (0.81, 1.53)	1.12 (0.95, 1.33)	1.00 (0.80, 1.26)
Minimum dietary diversity (MDD) for child (4 food groups)	–	1.17 (0.85, 1.61)	1.24 (1.05, 1.47)^a^	1.56 (1.24, 1.97)^a^
Minimum acceptable diet (MAD)	–	1.30 (0.88, 1.91)	1.28 (1.04, 1.57)^a^	1.25 (0.95, 1.64)

^a^statistically significant

Note: Multivariate logistic regression model predicting key IYCF knowledge and practices using intervention exposure: whether the mother saw any TV commercial or heard any radio spot from the ASTUTE intervention (not just those specific to nutrition). Each indicator estimate is derived from a multivariate logistic regression model that estimates the probability of the health indicator given exposure to the intervention. All model estimates control for maternal age, maternal education level, and wealth index. The “No Exposure” group refers to those who did not report any interpersonal communication (IPC) or media exposure

Knowledge of when to initiate breastfeeding was significantly lower for those who had IPC only or IPC plus media exposure compared to those with no behavior change communication exposure. Inversely, MAD was significantly higher for those with IPC only or IPC plus media exposure compared to those with none.

## Discussion

### Discussion

This study examined the impact of a comprehensive capacity-building and communication campaign designed to improve IYCFP in Tanzania. Results indicated that households in the study area generally experienced improved IYCFP knowledge and behaviors over time. Further, households with mothers and male heads of household who heard nutrition-specific communication messages through mass media were often associated with higher IYCFP. Lastly, overall exposure as measured using general ASTUTE messages and IPC through clinics and other similar venues was not consistently associated with higher levels of IYCFP.

#### Radio exposure

Mothers who remembered hearing at least one IYCFP radio ad were more likely to provide timely CF, provide MMF, provide MDD for the child and provide MAD, when compared to mothers who did not hear a radio ad. This positive association is consistent with other studies that report a 14.7% increase in MMF, 16.3% increase in MDD, and 22% increase in MAD after being exposed to both IPC and a media campaign  [19]. MAD showed the greatest association with mother’s radio exposure. In contrast, MMF practices showed the smallest association. A similar study conducted in Ethiopia reported that mothers who recalled hearing at least three radio spots had 1.06 and 2.9 times the odds of achieving MMF and MAD [20]. The Ethiopian study, however, deviates from the current study’s findings in that MMF was not associated with media exposure in Ethiopia [20]. Interestingly, mothers who were exposed to a radio ad were less likely to know when to initiate breastfeeding compared to mothers who did not hear a radio ad. This finding was inconsistent with a similar study which reported an increase of 3.01 in the mean regression score and an 8.5% increase in mothers from the intervention group who knew when to initiate breastfeeding [21, 22]. This result is unexpected and could be due to a confounder that was not accounted for in the study. One potential confounding factor is inadequate promotion of early initiation of breastfeeding at health facilities and among traditional birth attendants. Radio exposure among male heads of household varied slightly, with fewer significant associations.

When the male head of household recalled hearing a radio ad, mothers had greater odds of knowing when to initiate breastfeeding, and were more likely to achieve timely initiation of breastfeeding compared to when the male head of household had not heard a radio ad. No other studies were found that related a male head of household hearing an ad to the mother’s knowledge of when to initiate breastfeeding and achieving timely initiation of breastfeeding.

#### TV exposure

Mothers who saw at least one TV ad were more likely to feed their child a MAD, achieve MDD for their child, and know when to begin CF, compared to mothers who did not recall seeing a TV ad. Other studies had similar results, such as a 44.78% increase in CF knowledge among tribal mothers in India after viewing a video related to breastfeeding and CF practices [23], an increase in complementary feeding practices for mothers who were exposed to mass media [19], and a 2.7% increase in knowledge about when to initiate CF [22]. Another study in India found that exposure to a video about IYCFP was associated with an increase in MDD by 6.67% [23]. Furthermore, mothers were more likely to achieve MAD and provide MDD for their child when the male head of household had seen at least one TV ad compared to mothers with a male head of household who did not see a TV ad [23]. Not all studies have found positive significant associations between TV ads and IYCFP. A study in Bangladesh found exposure to TV ads did not significantly impact the amount of MAD and MDD in the population [19], though this trend was not true of radio ad exposure.

#### Mass media exposure

The value of mass media campaigns has been demonstrated in previous studies [24–26]. A large-scale television campaign in Vietnam reported that exposure increased a mother’s likelihood of exclusive breastfeeding and concluded that mass media should be part of a comprehensive program [26]. Another review article analyzed the outcomes of various mass media campaigns and determined that mass media does have the capacity to influence positive behavior changes while preventing negative changes which impact health on a large scale [24]. Our results are consistent with these findings. While media exposure was not positively associated with improvements among all IYCFP, TV and radio exposure was generally associated with several important changes in IYCF knowledge and behaviors.

### Limitations

A few limitations need to be considered when generalizing the results from this study. Most importantly, this study did not include a control group. While comparisons over time showed positive trends, respondents or communities without the intervention would have been helpful in controlling for a host of other possible influencing factors. Hence, much of the analysis considered self-reported intervention exposure. Additionally, since the ASTUTE program implemented various social behavior change campaigns apart from this behavior change communication, other program elements may have played a role in impacting IYCFP. While still subject to several limitations, the current study benefited from a robust sample and strong measures with results suggesting the intervention was associated with a number of positive outcomes.

### Future research

Future IYCFP research would benefit from strong study design strategies. Further, future studies should evaluate cost considerations associated with mass media and IPC campaigns to assess which elements are the most cost effective.

## Conclusion

The findings from this study suggest that a large, integrated behavior change communication inclusive of strategic communication strategies and approaches may help improve key child health behaviors. These findings further reinforce the value of targeted mass media campaigns which use TV and radio to influence health. Ultimately, the behavior change communication in Tanzania provides evidence to support similar comprehensive campaigns to reduce undernutrition and poor health outcomes in developing settings.

## Data Availability

The data that support the findings of this study are available from IMA World Health but restrictions apply to the availability of these data, which were used under license for the current study, and so are not publicly available. Data is however available from the authors upon reasonable request and with permission of IMA World Health. Contact Benjamin Crookston if you would like to request data from this study.

## Abbreviations

ASTUTE: Scaling up Growth: Addressing Stunting in Tanzania Early (in the under 5’s)
IYCFP: Infant and young child feeding practices
CF: Complementary feeding
MMF: Minimum meal frequency
MDD: Minimum dietary diversity
MAD: Minimum acceptable diet
IPC: Interpersonal communication
IMA: IMA World Health
DNuOs: District Nutrition Offices
CMSCN: Council Multisectoral Steering Committees for Nutrition
HLSCN: High Level Steering Committee on Nutrition
CHWs: Community health workers
SCT: Social cognitive theory
PDAs: Personal digital assistants
DMI: Development Media International
WHO: World Health Organization
IYCF: Infant and young child feeding practices
